# A combined method for correlative 3D imaging of biological samples from macro to nano scale

**DOI:** 10.1038/srep35606

**Published:** 2016-10-19

**Authors:** Manuela Kellner, Marko Heidrich, Raoul-Amadeus Lorbeer, Georgios C. Antonopoulos, Lars Knudsen, Christoph Wrede, Nicole Izykowski, Roman Grothausmann, Danny Jonigk, Matthias Ochs, Tammo Ripken, Mark P. Kühnel, Heiko Meyer

**Affiliations:** 1 Institute of Functional and Applied Anatomy, Hannover Medical School, Hannover, Germany; 2 Biomedical Research in Endstage and Obstructive Lung Disease Hannover (BREATH), Member of the German Center for Lung Research (DZL), Hannover, Germany; 3 Biomedical Optics Department, Laser Zentrum Hannover e.V., Hannover, Germany; 4 REBIRTH Cluster of Excellence, Hannover Medical School, Hannover, Germany; 5 Institute for Pathology, Hannover Medical School, Hannover, Germany; 6 Department of Cardiothoracic, Transplantation and Vascular Surgery (HTTG), Hannover Medical School, Hannover, Germany

## Abstract

Correlative analysis requires examination of a specimen from macro to nano scale as well as applicability of analytical methods ranging from morphological to molecular. Accomplishing this with one and the same sample is laborious at best, due to deformation and biodegradation during measurements or intermediary preparation steps. Furthermore, data alignment using differing imaging techniques turns out to be a complex task, which considerably complicates the interconnection of results. We present correlative imaging of the accessory rat lung lobe by combining a modified Scanning Laser Optical Tomography (SLOT) setup with a specially developed sample preparation method (CRISTAL). CRISTAL is a resin-based embedding method that optically clears the specimen while allowing sectioning and preventing degradation. We applied and correlated SLOT with Multi Photon Microscopy, histological and immunofluorescence analysis as well as Transmission Electron Microscopy, all in the same sample. Thus, combining CRISTAL with SLOT enables the correlative utilization of a vast variety of imaging techniques.

Correlative microscopy enables the combination and interconnection of results from different imaging techniques which allows a more comprehensive investigation of specimens and might lead to new insights in biomedical research^1^,^2^,^3^,^4^,^5^. Thereby, especially Correlative Light and Electron Microscopy (CLEM) proved to be useful by joining different resolution regimes as well as contrast mechanisms. Recent developments in the field of light microscopy techniques have pushed the limits to achieve higher optical resolution^6^,^7^, higher detection efficiency^8^,^9^, larger sample size^10^,^11^,^12^, shorter measurement duration^13^,^14^ and larger number of contrast mechanisms^15^. The utilization of new optical imaging techniques in the context of correlative microscopy is therefore a most promising perspective.

However, correlation often fails owing to various reasons: First, movement and deformation during the measurement as well as biological degradation of the sample lead to severe artifacts regarding specimen morphology and imaging data. Thus, results that are obtained with different imaging techniques are often hardly comparable. Second, there are conflicting requirements of the sample preparation as e.g. optical clearing^16^,^17^,^18^,^19^,^20^,^21^ for three-dimensional (3D) light microscopical investigation of large samples and rigid embedding for histological sectioning. The latter requires a transition of the sample into a different medium e.g. from the clearing solution into embedding material for subsequent cutting and bright field analysis^11^,^22^. This, however, is very time consuming and may lead to severe deformation of the sample inhibiting sufficient correlation. Third, it is nearly impossible to define distinct reference points to correlate structures from differing imaging techniques without a tool to bridge the gap between them^5^,^23^. This is essential for the correlation itself and for the design of the experiment e.g. decisions on further analyzing steps like the direction of histological sectioning or locating regions of interest (ROI) within the sample. In diseases like cancer, fibrosis or bacterial/parasite infection (e.g. tuberculosis) the ROI might be fairly limited in number and difficult to locate in the 3D environment of an affected organ. Therefore, the correlative combination of large-scale imaging techniques together with high resolution techniques enables an optimal evaluation of samples.

Here we use Scanning Laser Optical Tomography^8^,^11^,^24^ (SLOT) to image the entire accessory lobe of a rat lung with a resolution reaching down to the sub-alveolar level. SLOT is a highly efficient 3D imaging technique enabling simultaneous acquisition of absorption and fluorescence of specimens up to several millimeters. The 3D data sets generated with SLOT provide a holistic representation of the entire sample and allow for arbitrary virtual sectioning. Thus, distinct structures within the sample can easily be linked with findings from other optical microscopy techniques, thereby allowing easy achievement of correlative analysis. To address the need for deformation-suppressing rigid embedding and optical clearing of the sample at the same time, we developed the resin-based sample preparation method CRISTAL (***C**uring **R**esin-**I**nfiltrated **S**ample for **T**ransparent **A**nalysis with **L**ight*). CRISTAL infiltrates and encapsulates the entire specimen within a solid and transparent plastic resulting in an optically cleared biological sample, which is accessible for a large variety of imaging techniques. In combination with SLOT, it allows us to consecutively apply various imaging techniques to one and the same sample and correlate the results with each other. In addition to SLOT, we applied Multi Photon Microscopy (MPM) and Transmission Electron Microscopy (TEM) to the same sample. The measurements of these highly sophisticated techniques strongly differ in terms of resolution, contrast mechanism and field of view. They were accompanied by the analysis of histological and antibody stained sections of the same sample, all correlated with each other by SLOT.

We demonstrate the potential of this correlative imaging method utilizing SLOT and CRISTAL by initially presenting its application to a fibrotic lung lobe of a bleomycin-treated rat followed by a comparison of the results with correlative measurements of a second healthy control lung lobe.

## Results

### Simultaneous clearing and embedding of biological samples

We developed and applied CRISTAL to generate an optically cleared specimen entirely encapsulated within a solid and transparent cylinder made of UV-cured monomer. Optical clearing is a preparation step prior to optical microscopy, which suppresses the effects of light scattering. Dependent on the applied imaging technique and the optical properties of the tissue, many mesoscopic samples require optical clearing^16^,^21^,^25^.

**Figure 1:**
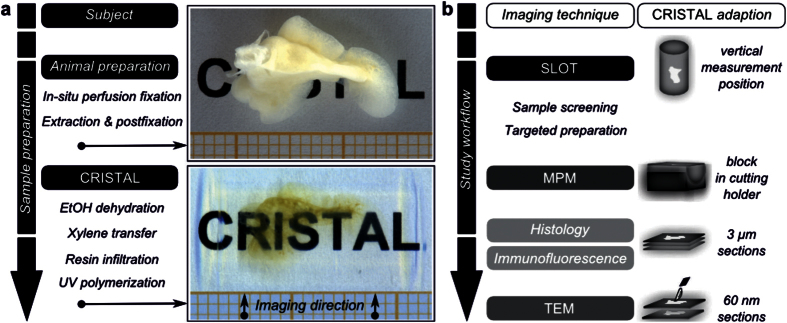
CRISTAL sample preparation and workflow of the correlative study. (**a**) Sample preparation steps of a rat lung lobe with the CRISTAL method. The accessory lung lobe of the rat is shown after fixation and after the resin-based clearing. (**b**) Workflow of the correlative study. First, the CRISTAL sample was screened by using SLOT. Based on the identified ROI, further investigations like MPM, histology or TEM were carried out. The CRISTAL block was adapted to the requirements of the various corresponding imaging techniques and sectioned for histology and immunofluorescence as well as TEM.

CRISTAL is based on the infiltration of a biological sample with a liquid monomer, which is subsequently cured by UV light to produce a specimen that is optically cleared and encapsulated in a transparent polymer (Fig. 1a). The CRISTAL procedure involves many of the same preprocessing steps as our previous described clearing technique based on MSBB^11^ e.g. *in situ* perfusion fixation, extraction and post fixation followed by dehydration with ethanol. Xylene is used to achieve miscibility with the fluid monomer, which is an individual mixture of two optical adhesives. The selected mixing ratio determines the refractive index *n* of the monomer and thereby *n* of the polymer. This is important to achieve optimal optical clearing. Here, for sufficient clearing of the rat lung, the polymer has *n* = 1.556 originating from *n* = 1.523 of the monomer. To contain the specimen during polymerization, we used a syringe giving the resulting CRISTAL sample a cylindrical shape with a smooth surface (Supplementary Fig. 1). The CRISTAL sample is completely encapsulated, appears optically clear in a wide spectral range (Supplementary Fig. 2) and is immediately available for a variety of microscopical investigations (Fig. 1b). For perfect oil immersion, we used an individual mixture of two silicon oils with the refractive index of the polymer.

### 3D Imaging at full scale with SLOT

**Figure 2:**
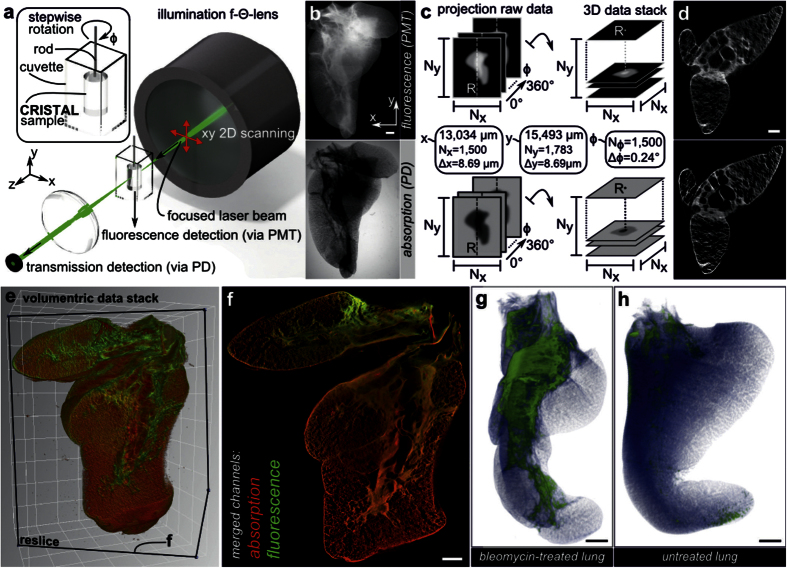
SLOT imaging and screening for ROI. (**a**) Volumetric imaging of the whole lobe with SLOT. The CRISTAL sample is immersed in a cuvette filled with immersion oil and mounted to a stage allowing for stepwise rotation. An f-theta objective forms a focused laser beam that is scanned through the sample to generate 2D projection images. Synchronized with the scanning process, transmitted light is recorded with a photo diode (PD), while fluorescence is simultaneously measured from below via photomultiplier tube (PMT). (**b**) Projection images of the lobe from one viewing angle, each covering a region of 13 × 15.5 mm. (**c**) By measuring a full revolution of the sample at step angles of 0.24°, two projection data sets with fluorescence and absorption data from 1,500 viewing angles are acquired. In order to generate 3D data stacks containing the whole sample, filtered back projection algorithms are applied to both sets. (**d**) Single tomograms after reconstruction of fluorescence and absorption channel. (**e**) Rendered volume overlay of absorption (red) and fluorescence (green) channels. (**f**) Reslice through the stack at the cutting plane indicated in **e** demonstrates alveolar resolution at different depths. Typical phenotype of fibrosis like parenchyma tissue thickening is visible. (**g**,**h**) Identification of ROI with increased tissue remodeling and collagen deposits is achieved by segmentation. Here, fibrotic regions (green) and normal parenchyma (blue) in the bleomycin-treated lung can easily be identified and compared with the untreated control lung. Scale bars, 1 mm (**b,f,g,h**), 500 μm (**d**).

We used SLOT to image entire CRISTAL samples (Fig. 2a). To suppress aberrations at the surface, the sample was mounted in a glass cuvette filled with immersion oil. For macro scale imaging of spacious samples, whose dimensions are on the length scale of centimeters, we chose a telecentric f-theta objective reaching a usable field of view of up to 45 × 45 mm. Fluorescence light was collected from the bottom of the cuvette and guided towards the detector via fiber bundle. Refinement of the data processing prior to image reconstruction eliminated artifacts in the tomograms caused by non-parallel beams in the xz-plane. This enables large scale 3D imaging of the entire sample covering a volume of 13 × 13 × 15.5 mm^3^ (Fig. 2c and Supplementary Video 1) with a voxel sampling of 1,500 × 1,500 × 1,783.

For complete spatial coverage of the lung lobe at alveolar level, we recorded 1500 projection images in a full revolution at an angular increment of 0.24° with an integration time of 5 s for each projection. Thereby a sampling rate of 8.69 μm per pixel in the xy-plane and a theoretical focal beam thickness of 20 μm (FWHM) were achieved. The latter is about one-third of the average diameter of an alveole in the rat lung^26^ assuming a spherical alveolar shape. We acquired projection data sets of autofluorescence and transmitted light containing information about absorption within the sample (Fig. 2b). After pre-processing, volumetric data sets are generated using a fast tomogram reconstruction that applies filtered back projection algorithms on graphical processing units (Fig. 2d). The resulting volumetric data stacks (Fig. 2e) are available for generating user-defined reslices, i.e. calculated sectional views in the 3D data, in any preferred direction (Fig. 2f). Distinct imaging planes and sections from other techniques were correlated by retrieving structure positions in SLOT data stacks. By comparing the bleomycin-treated disease model for fibrosis with the control lung lobe, patchy remodeled fibrotic tissue can be localized with SLOT. For further investigations the 3D spatial distribution of fibrosis in the lung lobe characterized by increased parenchymal tissue thickening caused by excessive connective tissue formation, here, the thickening of parenchyma tissue, can be determined by segmentation (Fig. 2g,h).

### Revealing details with MPM

**Figure 3:**
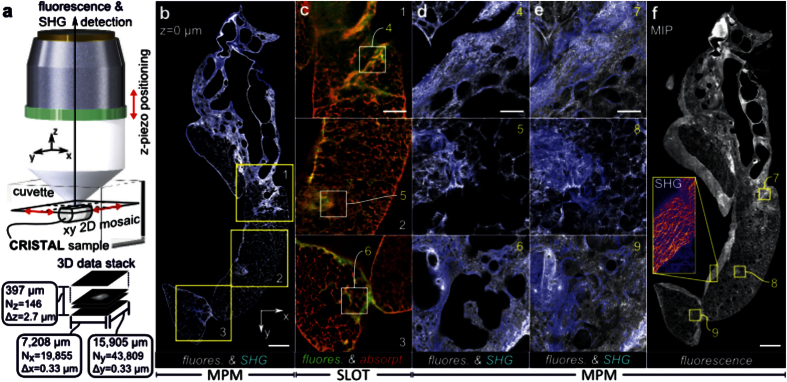
Revealing details with MPM and correlation with SLOT data. (**a**) Volumetric MPM imaging of the CRISTAL sample. After cutting for histological sections, the remaining CRISTAL block is immersed in a cuvette. The plane surface is positioned parallel to the xy-detection plane of the objective. By scanning ultra-short laser pulses, two-photon (2P) excited fluorescence and SHG are recorded simultaneously. In order to generate large volumetric stacks at high resolution, the CRISTAL sample is translated in the xy-plane for 2D mosaicing and the objective is positioned in the z-direction to measure at deeper layers. Thereby, a volume of 7 mm × 16 mm × 397 μm at subcellular resolution level is captured. (**b**) 2D MPM mosaic image showing 2P excited fluorescence and SHG (blue) in a complete slice through the accessory lobe on the surface of the CRISTAL block. (**c**) Corresponding reslices from SLOT data showing the correlation of fluorescence (green) and absorption (red) channel at the boxed regions in **b**. (**d**) Enlarged views from **b** at positions indicated with boxes in **c** shows the alveolar architecture at subcellular resolution. (**e**) Overlay of maximum intensity projections (MIP) from SHG and fluorescence channel through whole volume depth of the regions shown in **d**. (**f**) MIP of fluorescence channel through the complete volume demonstrating the stability and potential of the long term MPM measurements. The boxed MIP of SHG visualizes collagen fiber structures within the pleura. Scale bars, 1 mm (**b**,**f**), 500 μm (**c**), 100 μm (**d**,**e**).

The CRISTAL sample was cut to generate a truncated resin block offering a plane. MPM was performed using an upright setup equipped with an automated high precision x-y-translation table (Fig. 3a). The objective was a 20× water-immersion lens with an NA of 1.0 and a working distance of 1.8 mm mounted to a piezo-driven z-translation stage for axial sectioning.

We recorded 3D mosaics covering a volume (x, y, z) of 6,515 × 14,965 × 397 μm^3^ using tiles with a size of 350 × 350 μm and 0.3295 μm lateral resolution at 0.8 s per tile (Fig. 3b) in z-steps of 2.72 μm. This corresponds to 20 × 46 = 920 tiles and 12.5 min scanning duration for one complete 2D slice. Lateral positioning of the sample was performed by the xy-table with an overlap of 5% while z-translation was realized by the objective stage. We measured autofluorescence at 525 nm ± 25 nm excited at 850 nm by two photon absorption and back-scattered second harmonic generation (SHG) signal at 425 nm (Fig. 3d). The theoretical lateral optical resolution was 0.3 μm. Custom macros were utilized for subsequent data stitching (see Online Methods). Starting at the cut surface of the CRISTAL block, we observed signal decay at deeper layers due to material mismatch, since the objective is designed for water-based samples in contrast to glass-like samples like CRISTAL. However, a maximum intensity projection (MIP) of fluorescence and SHG signal along the z-axis (Fig. 3f) visualizes morphological und functional fine structure throughout the measured volume at subcellular resolution level (Fig. 3e). In comparison with fixed lung samples filled with air^27^,^28^,^29^, identical patterns of elastin via autofluorescence and collagen via SHG are observable. In general, endogenous tissue components like elastic fibers as well as the different lung cell types (parenchymal and non-parenchymal) are visualized over the whole lung organ (Fig. 3b,d,e and Supplementary Fig. 3). Due to the function of elastin as a structure- and elasticity-lending component, autofluorescence is present in the whole lung with an emphasis in blood vessels, bronchial walls and especially in the fibrotic remodeled areas of the disease model. SHG visualizes predominantly the rather inelastic tensile strength collagen. Universally present in the alveolar lung tissue as well as particular in blood and conducting airway walls, collagen is typically increased in fibrotic regions caused by bleomycin treatment, too (Fig. 3b,d,e and Supplementary Fig. 3). Detailed analysis of the SHG signal in the MIP reveals a dense network of fiber-like threads within the pleura (Fig. 3f). By aligning the SLOT volume data to the MPM volume, correlative comparison between both stacks at any preferred region within the sample was possible (Fig. 3b–d). SLOT provides a general view on the entire accessory lobe and connects imaging results from different regions.

### Histological and immunofluorescence analysis of sections

**Figure 4:**
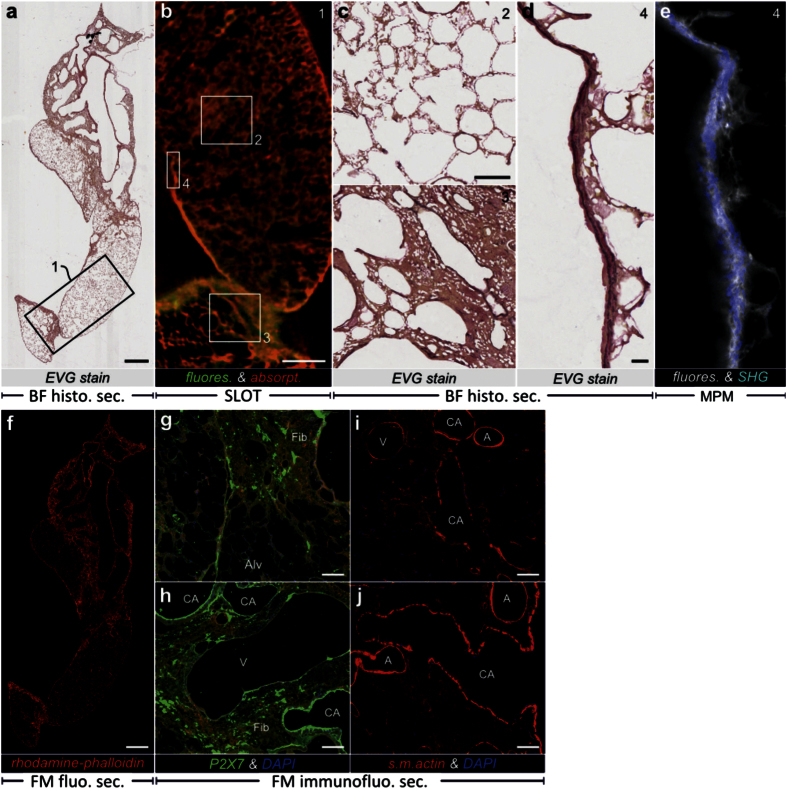
Histological and fluorescent sections of the CRISTAL sample in correlation with SLOT and MPM. (**a**) Bright field (BF) microscopy analysis of section from fibrotic rat lung stained with Elastica van Gieson (EVG) after MPM measurements demonstrating elastic fibers in the accessory lobe. (**b**) Corresponding reslice through SLOT 3D data showing correlation with fluorescence and absorption channel at the boxed region in **a**. (**c**) Enlarged views from **a** indicated by the squared boxes in **b** demonstrate differences between fibrotic (ROI 3) and non-fibrotic (ROI 2) tissue at subcellular resolution level. (**d**) Enlarged view showing the pleura region from the small box in **b** that corresponds to the fiber structures in the SHG signal from (**f**). (**e**) By correlating it with the corresponding MPM slice visualizing fluorescence and SHG in the CRISTAL sample, the origin of the SHG signal in (**f**) can be traced back to collagen fibers. (**f**) Fluorescence Microscopy (FM) detection of F-actin fibers in a CRISTAL section of a fibrotic rat lung lobe using rhodamine-phalloidin (red). (**g**,**h**) Immunofluorescence (IF) of the purinergic receptor P2X7 reveals intense staining of discrete regions within fibrotic remodeling areas as well as bronchial epithelia (green). (**i**,**j**) IF of smooth muscle actin in walls of conducting airways, blood vessels and individual cells in the fibrotic remodeled tissue as well as parenchyma (red). (Conducting airways (CA), veins (V), arteries (A), alveoli (Alv), fibrotic remodeling (Fib)). Scale bars, 1 mm (**a**,**f**), 500 μm (**b**), 100 μm (**c**,**g**–**j**), 20 μm (**d**,**f**).

**Figure 5:**
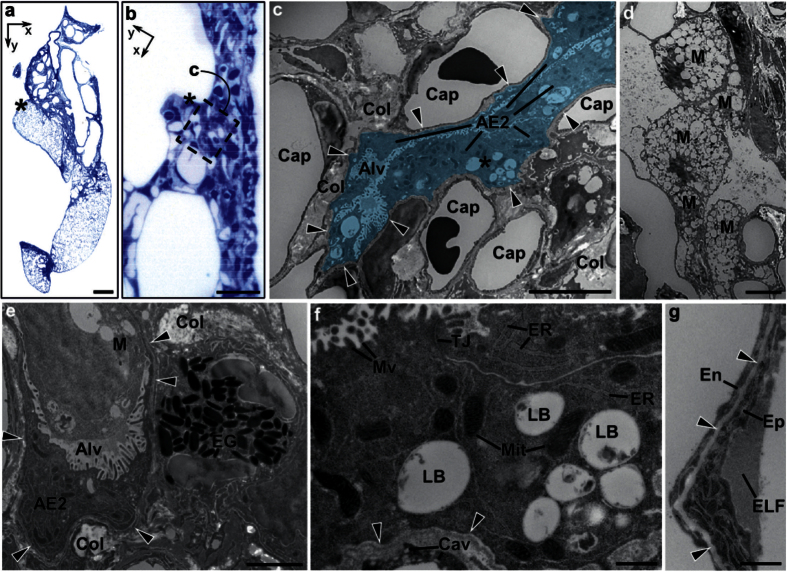
Correlative TEM analysis of SLOT selected areas in bleomycin-treated lung. (**a**) Histological section with Toluidine blue staining of targeted area in the lung. The TEM analyzed area is marked with asterisk and shown in a closer look (**b**) within a TEM correlation. (**c**) Electron microscopy image (area of **b**, box with asterisk) of lung parenchyma showing capillaries (Cap) and collapsed alveolus (Alv), highlighted in blue, with accumulation of alveolar epithelial type II cells (AE2). The alveolar epithelial basal lamina can be traced (arrow heads). Large depositions of collagen fibrils (Col) are visible. (**d**) Alveolus with several macrophages (M). (**e**) Detail of eosinophil granulocyte (EG), collagen depositions (Col), and collapsed alveolus (Alv) with alveolar epithelial basal lamina (arrow heads), alveolar epithelial type II cell (AE2) and macrophage (M). (**f**) High magnification of alveolar epithelial type II cell (area of **c**, asterisk). Subcellular details like ribosomes, endoplasmic reticulum (ER), mitochondria with cristae (Mit), apical microvilli (Mv), tight junction (TJ) and lamellar bodies (LB, lamella mostly extracted) are visible. On the vascular side of epithelial basal lamina (arrow heads), the caveolae (Cav) of the endothelial cell can be identified. (**g**) Blood-air barrier with endothelium (En), basal lamina (arrow heads) and epithelium (Ep) with protein-rich epithelial lining fluid (ELF). Scale bars, 1 mm (**a**), 10 μm (**b**), 5 μm (**c**,**d**), 2 μm (**e**), 0.5 μm (**f**,**g**).

We performed sectioning along the y-axis of the CRISTAL cylinder before and after MPM measurements. Elastica van Gieson (EVG) (Fig. 4a) and toluidine blue (Fig. 5a,b) as two standard histological stainings were probed. The toluidine blue staining provides an overview of the sample via dark blue staining of nuclei and light blue staining of cell bodies. Interstitial thickening, inflammation with increased cell infiltrates and alveolar collapse of the bleomycin treated lung can be detected compared to the untreated lung (Supplementary Fig. 4). Additionally, characteristic features of the conducting airways and blood vessels stratification can be clearly separated. EVG stained sections (Fig. 4a) show the typical staining pattern for lung samples. Collagens are bright red, elastic fibers violet-black, muscle tissue and cytoplasm yellow. By comparing the bleomycin-treated with the control lung, the typical patchy isolated collagen deposits with elastic fibers are visible especially under the pleura and close to the bronchus indicating fibrosis and parenchymal consolidation.

We checked for the orientation of the sections by correlating the EVG section with the aligned SLOT data stack and observed good agreement (Fig. 4b). Enlarged regions of the section were correlated with the corresponding SLOT reslice to demonstrate the relationship between thicker tissue structure and stronger autofluorescence signal in the SLOT measurements (Fig. 4c). Using a correlative analysis of the EVG stained sections with corresponding SHG signal from MPM measurements (Fig. 4d,e), we were able to identify collagen as the origin of the fiber-like fine structure in the lung pleura (inset in Fig. 3f).

In addition to histological (Fig. 4a,c,d) and fluorescent (Fig. 4f) staining, immunofluorescence detection of lung antigens was established (see Online Methods). Here, the purinergic receptors P2X7^30^,^31^, a ligand-gated ion channel, which is activated in inflammation and tissue damage (Fig. 4g,h) and the antigen detection of smooth muscle actin^32^ as a marker of myofibroblasts (Fig. 4i,j) is shown. An increased expression of both tested antigens was detected in the bleomycin treated lung compared to the control lung (Supplementary Fig. 6).

### Analysis with TEM

CRISTAL lung lobes were prepared for TEM using standard TEM equipment (see Online Methods). For correlative and targeted TEM, two adjacent slices were prepared for histology (Fig. 5a,b) and TEM (Fig. 5c–g), respectively. The observed samples show that previous treatment and resin embedding preserves the overall ultrastructure of the tissue and allows subcellular electron microscopic analysis. Structures and cell types show the typical TEM morphology and can be easily identified e.g. alveolar type I and type II cells (Fig. 5c–g and Supplementary Fig. 5). Alveolar type I cells, which cover over 95% of the alveolar surface, are seen with their typical spindle shaped cell body and protrusions lining the alveoli, together with alveolar type II cells. Cells show well preserved nuclei, with hetero and euchromatin, a surrounding double membrane, nuclear pores and nucleoli, preserved cristae structure and double membrane in mitochondria, endoplasmic reticulum, ribosomes, as well as tight junctions (Fig. 5f, Supplementary Fig. 5e,f). Even the lamellar bodies, known for their difficult preservation in TEM, can be identified, although the lamellae are mostly extracted. This is due to the deliberate missing of optimal lipid fixation with osmium and uranyl acetate, which would inhibit optical clearing. The blood-air-barrier shows the typical well conserved stratification of endothelium, alveolar epithelial basal lamina and epithelium (Fig. 5g, Supplementary Fig. 5g). The conducting airways have their typical conducting airway composition with respiratory epithelium, smooth muscle and surrounding collagen (Supplementary Fig. 5c). In comparison, the bleomycin-treated lung possesses increased amounts of collagen (Fig. 5c,e) as well as infiltration of macrophages and granulocytes (Fig. 5d,e). As already described in Lutz *et al.*^33^, blood-air-barrier shows the typical thickening and alveolar collapse (Fig. 5c labelled in blue).

## Discussion

By establishing and combining the sample preparation method CRISTAL with a SLOT setup specially designed for large scale 3D imaging, we achieved correlation between MPM, histology, immunofluorescence and TEM within entire biological samples covering volumes up to the range of cubic centimeters.

Since CRISTAL optically clears and rigidly embeds a biological specimen at the same time, it merges two types of sample preparation that were separated up to now. On the one hand, optical clearing procedures (e.g. Clarity^20^, Scale^19^, DBE^16^, BABB^10^ and MSBB^11^) permit 3D imaging in primary strongly scattering tissue but do not provide histological sectioning (3 μm) without a transfer in rigid substances. On the other hand, rigid embedding (e.g. technovit and paraffin or EPON, lowicryl and LRWhite for histology or TEM, respectively) allows thin sectioning of the specimen but generally leaves it nontransparent and therefore inaccessible for optical imaging at large scales. We showed that CRISTAL samples are accessible for 3D large scale imaging and thin sectioning without further steps. The principle of optical clearing is similar to that of the immersion method^32^, but employs a monomer curing to an index matched polymer instead of a refractive index matching liquid for infiltration. Thus, optical clearing and hardening of the sample starts with the initiation of the UV-curing. This, in contrast to resins with polymerization starter components, allows to control the process and to adapt the incubation time. CRISTAL works in a variety of different tissues like lung, brain, bone and cartilage offering a broad range of applications (Supplementary Figs 7 and 8). The achieved transparency allowed optical imaging of entire biological samples of centimeter thickness, which supports the assumption that CRISTAL is comparable to other clearing techniques^17^. In contrast to the control lung (Supplementary Figs 3 and 4) slight blurring remained in the bleomycin-treated lung. This indicates mismatches of the refractive index in the severely remodeled fibrotic regions, which are very compact and hamper infiltration. A CRISTAL embedded sample can be sectioned for histology as well as TEM allowing the correlation of 3D morphological data with standard tissue analysis. Toluidine blue and EVG show typical staining characteristics. For a more specific analysis, even immunofluorescence of individual target antigens can be performed. CRISTAL leaves the ultrastructure of the tissue almost completely intact. Only lamellar bodies showed extraction of the lamellae. Ultrastructure of cells and surrounding connective tissue is very well preserved and comparable to typical TEM embedding^34^.

In contrast to liquid clearing methods, CRISTAL embedded samples required no re-embedding after imaging in order to perform histological, immunofluorescence or TEM analysis. Time consuming and ultrastructure loss or deformation making transfers into sliceable blocks can be avoided. In addition it should be noted that ROIs can be identified in the 3D SLOT dataset and samples can be orientated accordingly prior to sectioning. As a consequence MPM and EM as wells as histological and immunohistochemical analysis of ROIs can be performed in optimal orientations. Current efforts to correlate light with electron microscopy had to deal with the re-embedding of the sample or perform light microscopy without clearing in standard TEM embedding resins, which means that 3D imaging is limited to only a few micrometers of depth^2^.

Optical 3D imaging techniques like Ultramicroscopy and SLOT make great demands on a sample in terms of optical properties, tissue deformation and imaging movement artifacts^11^,^35^. CRISTAL samples meet those requirements by providing transparent access in any direction due to the optical clearing. Individual mixtures of silicon oils allow uncomplicated immersion of the sample in a non-toxic and inert way. From the mechanical point of view, an embedded sample eliminates motion artifacts caused by movement and deformation of the specimen during the measurement. The application of 3D imaging techniques can be accompanied with sectioning of CRISTAL samples, allowing e.g. the direct comparison of mechanical slices with sections from MPM measurements. Due to the rigid embedding, long measurement durations and iterations do not pose a problem. Moreover, sectioning and 3D imaging can be spatially and temporally divided without noticeable artifacts allowing microscopy-accompanied serial block face methods^36^. However, cell tracking based on fluorophores, like GFP, is potentially difficult in CRISTAL embedded samples owing to denaturation of the protein structure via dehydration and UV exposure during the sample preparation. MPM measurements showed strong autofluorescence of the tissue but weak back-scattered SHG signal. The latter is due to the optical clearing, since SHG is a process producing light in the direction of transmission^34^. In contrast to the unfavorable water-immersion objective, a specially designed oil immersion objective with long working distance would significantly enhance quality and imaging depth of MPM measurements in CRISTAL samples.

The correlative comparison of reslices in the SLOT 3D stack with results from MPM, histological and fluorescent sections as well as TEM in arbitrary directions indicate the potential of SLOT as a tool for large scale imaging that bridges the gap between these methods. SLOT simultaneously provides access to absorption, scattering and fluorescence in entire biological samples covering a volume in the cubic centimeter range. This is a result of the described modifications applied to the original setup^8^ and the unique measurement principle that works without extensive detection designs. As a consequence SLOT is a well suited tool for prior screening of entire samples before further analyses. The correlative method presented here is based on the identification of distinct structures in the sample in order to align the volumetric SLOT data stack. Therefore, beside fine structures within the specimen itself, additional marker like beads or fibers embedded in the surrounding resin of a CRISTAL sample could be beneficial.

In summary the combination of SLOT 3D imaging with the CRISTAL sample preparation allows for a portfolio of state of the art imaging and analysis techniques and different contrast mechanisms to be combined consecutively on the very same sample enabling the correlation of results from macro scale down to the subcellular level (e.g. using sections, MPM or TEM) within samples at the cubic centimeter range (Supplementary Video 1).

The versatile compatibility and convenience of this approach thereby allows the correlative extension with additional analytical as well as optical imaging techniques in order to further broaden the portfolio of utilized methods in a study.

## Methods

### Animal husbandry

For this study, 10 weeks old male Fisher rats F344 from Charles River laboratories GmbH and 12 weeks old male C57BL/6 wild type mice were used. The animals were housed in an animal facility at Hannover Medical School and were provided with food and water *ad libitum*. The animal care was in accordance with the “Principles of Laboratory Animal Care” (National Society for Medical Research) and the “Guide for the Care and Use of Laboratory Animals” (NIH publication 85-23, revised in 1996) at any time. These protocols were approved by the National Society for Medical Research as well as the authorities of Lower Saxony, Germany.

### CRISTAL sample preparation

A detailed protocol of the animal treatment is given in Lutz *et al.*^37^. Briefly, in contrast to the control rat who did not receive any treatment, the treated rat was anesthetized by inhalation of 4% isoflurane in a Plexiglas chamber and intubated with a 14 GA catheter (BD Becton Dickinson). Afterwards, bleomycin (1.28 U/rat) was instilled intratracheally to both lungs of the rat. The used dosage of bleomycin was selected to induce fibrosis under consideration of minimizing mortality. At day 14 after bleomycin treatment the control and treated rat lungs were fixed *in situ* by using a two-stage flushing system. For this, the abdomen was opened, the inferior caval vein was cannulated and the blood vessel system was flushed at a pressure of 30 cm liquid column with a NaCl solution (B. Braun Melsungen AG) including 0.5% heparin (Ratiopharm GmbH). During the inflation of the lungs via a tracheal intubation with an inflation pressure of 10 cm liquid column, lungs were fixed with a mixture of 0.1% glutaraldehyde and 4% formaldehyde in 0.2 M HEPES buffer in the same way as the first flushing. Later, lungs were extracted and post-fixated for 6 h at 4 °C.

In order to generate CRISTAL samples, whole lungs were dehydrated through 2 h incubation steps at 4 °C in an increasing ethanol series (J.T. Baker) of 30%, 50%, 70%, 90% and twice 99.8% followed by an increasing xylene (1,2-dimethylbenzene) series (J.T. Baker) of 50%, 70%, 90% and twice 100%. Thereafter, lungs were transferred with an incubation time per step of 1 day at room temperature in an increasing resin (NOA 68 and NOA 71, both from Norland Products Inc.) mixture series of 50% and twice 100%. Only the combination of sufficient dehydration and correct refrative index adjustment ensure the successful clearing. Due to slightly variations in equilibration characteristics of different tissue samples, especially final concentration of 99.8% ethanol, 100% xylene and 100% CRISTAL must be checked individually. Here, additional solution changes are required until no smear formations are observable any longer. The refractive index of the resin mixture monomer, which includes a combination of NOA 68 and NOA 71, was adjusted to 1.523 with an Abbe refractometer (Müller Optronic) through the mixture of the two input resins with a ratio of approximately 1:7 to obtain a refractive index of the resin mixture polymer of 1.556. Due to slight differences per product charge and storage period, the refractive index had to be checked individually with the refractometer. To avoid scattering inside the sample through residual gas bubbles, samples were placed under low pressure in a vacuum chamber for several days. Due to the projection-based measurement principle of SLOT, a smooth and cylindrical surface of the CRISTAL sample is beneficial. Therefore, we used disposable syringes as blank molds (Supplementary Fig. 1). Lungs were mounted on spacers in a syringe filled with the resin mixture. The upper half of the resin was allowed to polymerize by alternating 1 min UV light exposure (range 374 nm to 383 nm) and 1 min cooling steps for 30 min at 4 °C. After that, the syringes were turned upside down, the spacers were removed and the entire volume of the specimen was allowed to polymerize with increasing UV light intensity for a total time of 24 h. After the polymerization the syringes were removed and the final CRISTAL samples, optically cleared and embedded specimens in polymerized resin, were mounted on the rotation stage of the SLOT system. Regarding clearing of other types of biological samples, resin incubation times have to be adapted (brain: 3 weeks, bone: 2 weeks) and minor changes of the refractive index might be necessary dependent on the tissue.

### Large scale 3D imaging with SLOT

For the successful 3D imaging of samples at the macro scale i.e. spanning several millimeters in all dimensions, the original SLOT setup^8^ had to be modified in several points: The laser light of 532 nm and 635 nm laser diodes in continuous wave mode is delivered via a single mode fiber (P3-460A-FC-1, Thorlabs) to a motorized zoom lens (H10Z1218MP, computar). The latter generates a collimated laser beam with a defined beam diameter which is coupled into a 2D xy-galvanometer scanning cube (ProSeries II Scan Head, Cambridge Technology) with a maximum aperture of 14 mm. Thereby, the slow y-scanning mirror deflects along the y-axis while the fast x-scanning mirror meanders in the direction of the x-axis (see Fig. 2a). The laser beam is scanned through a telecentric f-theta scanning lens (S4LFT0080/121, Sill Optics) that allows a field of view of up to 45 mm in diameter. This is essential for the measurement of samples in the millimeter range. The f-theta lens is positioned with the y-scanning mirror of the scan cube in its back focal plane. Thus, focused laser beams in the yz-plane are parallel to each other, i.e. the beams are all orthogonal relative to the axis of rotation. In the xz-plane, however, the focused laser beams tend to form a fan-like shape that has to be compensated for before the reconstruction process (see below). That way, the weakly focused laser beam is scanned through the sample, which is mounted to a rotation stage (M-060.PD, Physik Instrumente) and immersed in a glass cuvette (700.016-OG, Hellma Analytics) filled with a custom mixture of two silicon oils (OI-808-2 and OI-808-5, Onlink Technologies, equivalent to DC 702 and DC 705, Dow Corning). To suppress refraction and reflections, its refractive index is adjusted so that it matches the refractive index of the sample, i.e. the cured mixture of polymer in the CRISTAL sample with n = 1.556. Thereby, the numerical aperture (NA) of the illumination optical system is determined by the beam diameter that is adjusted using the motorized zoom lens in front of the scan cube. The NA is chosen depending on the size of the sample, i.e. its thickness in the xz-plane, since in SLOT the sample has to be covered by the depth of field DoF = 2 × *n *× λ/NA^2^ of the illumination laser beam. Here *n* is the refractive index of the immersion medium and *λ* is the laser wavelength. We scanned the accessory lobe of the rat lung with an NA of 0.013 resulting in a DoF of approx. 10 mm and an optical resolution of Δ*x* = *λ/*(2NA) = 20 *μm*. Two projection images were generated by simultaneously recording transmitted and fluorescence light at 1,500 × 1,780 sampling points with 15 bit pixel depth in 5 s per viewing angle. By rotating the sample in steps of 0.24°, two projection data sets were measured resulting in 1,500 projection images per set. This takes into account the sampling theorem that requires the number of projections to be equal to the number of sampling points of the x-axis known from computed tomography^37^.

In SLOT, light from the sample only needs to be collected and sequentially measured without producing an image on a 2D detector, e.g. a CMOS sensor. Since the origin of transmitted and fluorescent light is intrinsically known due to defined scanning of the focused illumination beam, the main task is to homogenously collect and guide light from the sample to a 1D detector, e.g. a photo diode (PD) or a photo multiplying tube (PMT). This can easily be achieved by simple optics design even for larger sample volumes. For the detection of the transmitted light behind the sample, we used a standard 2″ plan-convex lens and an amplified PD (PDA36A, Thorlabs). A diffusor in between compensates for back reflected light from the PD’s surface. For the collection of fluorescent light we used a custom-made fiber bundle optically coupled to the bottom of the glass cuvette. The other end of the bundle has a rectangular shape to match the shape of the PMT's sensitive area (R3896, Hamamatsu). Thus, by performing a 1:1 imaging of the fiber bundle with two aspheric condenser lenses, an efficient utilization of the PMT is achieved. Scattered laser light was blocked with a 570 nm long pass filter (FGL570, Thorlabs).

Controlling and data acquisition was performed using custom software written with C++ and QT framework on a personal computer equipped with digital acquisition hardware (NI PCI-6221 and NI PCI-6251, National Instruments). Automation of the filter wheel and the translation stages was achieved by utilizing microcontroller boards (UNO, Arduino). After successful acquisition, the projection data sets are processed with a PC equipped with a high-end graphical processing unit (GPU, GeForce GTX 590, Nvidia) and 32 GB memory. In order to perform parallel beam reconstruction, the projection data needs to be preprocessed to compensate for the fan-beam in the xz-plane as described above. Therefore, a custom macro in the open source software ImageJ^33^ is applied, that removes shearing along the φ-axis in the sinogram representation of the acquired projection data. Thus, sinus-like functions are regained that correspond to a parallel propagation of the focused laser beams in the sample. Processed in that way, projection data is reconstructed (see Fig. 2c) with the program *tilt* from the open source tomography environment IMOD by applying the filtered back projection (FBP) on GPU. Subsequent image processing steps for correlation purposes are describe in the section “data alignment”.

The volumetric 3D rendering of 3D SLOT data stacks (see Fig. 2e) was performed with the open source software voreen (http://voreen.uni-muenster.de). Both data sets from PMT and PD detection were loaded and combined in voreen with adjusted transfer functions for best visibility of autofluorescence (green) and absorption (red) channel. The coordinates of the introduced clipping plane were extracted and applied in ImageJ in order to obtain a reslice through the SLOT data set at the desired plane (see Fig. 2f).

The segmentation-based screening of the lung samples (see Fig. 2g,h) was carried out by a semi-automatic adaptive brush tool in ITK-snap^38^ (www.itksnap.org) based on ITK (www.itk.org) and VTK (www.vtk.org). Due to their higher density compared to not altered tissue, fibrotic regions can be segmented and therefore ROIs defined by applying a gradient-anisotropic-diffusion and gradient-magnitude image filter as well as a watershed tool.

### Two photon and SHG imaging of large samples

Two photon and second harmonic generation (SHG) measurements of the rat lung CRISTAL samples were performed with an upright Multi Photon Microscopy setup (TriM Scope II, LaVision BioTec) supplied with ultra-short laser pulses of a Ti:Sa femto-second oscillator (Chameleon Ultra II, Coherent). In order to measure a volume deep within the lung, the originally cylindrical CRISTAL sample was cut prior to measurements with MPM (see part on sectioning for details). The cutting direction was chosen nearly parallel to the cylinder’s axis of rotation resulting in a truncated cylinder block offering a plane surface on one side. Due to the prearrangements of the sectioning, the cylinder is glued to a rectangular glass block for mounting purposes. Utilizing this glass block, the CRISTAL sample was mounted to a kinematic sample holder (KM100PM/M, Thorlabs) which was fixed on a precise xy-translation stage (Motorized xyz-stage, LaVision BioTec) in order to align the cut surface parallel to the xy-translation plane. The objective (W Plan-Apochromat 20×/1.0 DIC M27 70 mm, Zeiss) was chosen for its long working distance of 1.8 mm in combination with a NA = 1.0 and a magnification of 20×. However, it is optically corrected for water-immersion instead of the ideal glass-like refractive index immersion required for CRISTAL samples with n = 1.556. Unfortunately, such microscope objectives are currently not available. Due to the required water-immersion of the objective and the large measurement area of about 114 mm^2^, we decided to completely immerse the mounted CRISTAL sample in a water bath. As we had experienced negative effects of water that had been drawn into cut CRISTAL samples during preparation development, we sealed the plane surface with a thin layer (approx. 200 μm) of the same polymer mixture after sectioning and before MPM measurements. Following, previously observed opacity formations caused by water entry into the sample did not occur.

After the sample alignment, a 2D mosaic was defined covering the whole sample with 20 × 46 tiles of 350 × 350 μm size and 1,062 × 1,062 pixels resulting in a sampling resolution of 0.3295 μm per pixel. Every tile was scanned with 5% overlap between adjacent tiles. Thus, one measured 2D mosaic consisted of 920 tiles and 19,772 × 45,417 pixels resulting in 6,515 μm × 14,965 μm. We measured autofluorescence at 525/50 nm and SHG at 425 nm applying a laser wave length of 850 nm. The average power was adjusted from 50 mW to 40 mW starting at the deepest slice in the sample up to the top slice. Thereby the z-piezo driven position of the objective was displaced for every slice at steps of 2 μm, which corresponds to a measured offset of 2.72 μm due to the refractive index induced optical shift between water and the CRISTAL polymer. Thus, the 146 recorded slices correspond to a total measured depth of 397 μm. We observed no significant artifacts owing to instabilities of the setup, although the measurements lasted several days.

For the 3D mosaicing of all captured slices a total number of 134,320 tiles corresponding to 282.7 GB per channel had to be processed. Mosaicing was performed on a high-end office PC applying custom ImageJ macros: For stitching, linear blending from one to the next tile was introduced by a pre-weighting mask applied to every tile. Afterwards, every tile was copied and pasted into a prepared large scale image memory. The tile positions were calculated using two iteratively determined translation vectors accounting for the overlap between neighboring tiles and the tilt between the translation stage and optical scan-axes.

### Volumetric SLOT data alignment for correlative reslice analysis

A reslice is a calculated image representing a sectional view through a 3D data stack. Thus, a reslice is similar to a virtual cut through a sample volume and can have the shape of a flat or curved plane. Thereby, reslice calculation is based on interpolation of pixel values from the original pixel in the 3D stack that are nearest to the chosen plane. 3D data stacks obtained with SLOT are intrinsically oriented along the direction of the axis of rotation, i.e. the axis is orthogonal with respect to the tomogram planes. Also, since SLOT exhibits an isotropic optical resolution and sampling, virtual sectioning of the stack can be performed in any preferred direction without suffering image distortion. Therefore, the alignment of SLOT volumes to generate reslices corresponding to measurements from other imaging techniques for correlative analysis is a straight forward task. When correlating data sets from different techniques with SLOT measurements we will refer to the data sets from these other techniques as the “initial images”.

First, the 3D data has to be available in an environment allowing arbitrary reslicing and measurement of coordinates of points located on this reslices. Therefore, we chose the *volume viewer* plugin of ImageJ. After loading the SLOT 3D data stack, the whole volume needs to be manually tilted in a way that parallel reslices roughly comply with features of the initial image that was chosen for comparison. Then, in three regions of these reslices enlarged parts are compared and explored for smaller features that fit to the initial image. At these points xyz-coordinates of position vectors 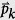 are taken.

Second, after measuring coordinates of distinct structures at 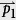, 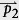 and 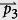 that lay in the initial plane, one can calculate the normal-form of the corresponding reslice plane through the SLOT 3D data stack. Therefore, two vectors 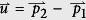 and 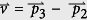 connecting the position vectors 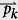 are set to subsequently calculate their normal vector 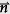 with 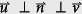 by applying the vector product 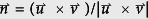. The normal vector 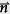 is oriented orthogonal to the desired slice through the SLOT 3D data stack that corresponds to the initial image. Thus, the points 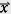 of the reslice can be described with 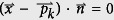 where 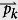 is one of the measured position vectors.

Third, in order to achieve alignment of both stacks, i.e. the SLOT data stack and the volumetric stack chosen for comparison, a transformation of the SLOT 3D data stack has to be performed. Therefore, the stack originally oriented along the axis of rotation is rotated so that 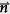 is orthogonal with respect to the coordinate plane, which is here spanned by 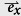 and 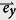. Thus, one has to calculate the angle of rotation along the x-axis 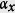 and the y-axis 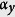 by solving 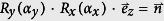 with the rotation matrices 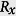 and 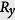. This yields 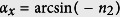 and *α*_*y*_ = acrsin (*n*_1_/cos (*α*_*x*_)). We applied the *transformJ* plugin in ImageJ to rotate both SLOT 3D data stacks from PMT and PD channel in order to obtain reslices parallel to the orientation of the comparative volumetric stack. Analogically, the initial image in this stack can be found by utilizing the rotated position vector 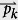 of the normal form.

These generated reslices correspond to flat sectional views through the volumetric stacks. In the special case of curved reslices (Supplementary Fig. 4) virtual sectioning is performed along a line following manually adjusted intersection points. Therefore, we aligned the SLOT data stack according to the procedure described above and subsequently applied the *Dynamic Reslice* plugin in ImageJ to manually retrace the cutting line of the corresponding section.

### Histological sectioning

Serial sections (3 μm) from the lungs previously analyzed by SLOT and MPM were stained by toluidine blue and Elastica van Gieson’s Stain (EVG) for conventional histopathological assessment of the fibrotic as well as healthy control tissue. Protocols of toluidine or EVG staining were used for histology with following modifications:

For toluidine staining, slides were incubated 2 min in toluidine blue, were washed for 1 min in distilled water and were subsequently transferred in an increasing ethanol series starting with 70% ethanol until 100% ethanol. Before EVG staining slides were slightly shallow etched with half-saturated sodium hydroxide solution for 20 min. After that, histological slides were incubated 30 min in elastin solution, followed by 15 min Weigert’s-solution and 1 min picrofuchsin solution with several distilled water washing steps of 1 min in between. Finally, slides were transferred in an increasing ethanol series such as the toluidine staining. In both stainings histological slides were air-dried, embedded with the clearing resin mixture (polymer n = 1.556) and UV polymerized for 1 h. Histological sections were recorded at a 10× magnification with a Zeiss AxioScan.Z1.

### Antibody staining

For the detection of specific markers of lung tissue, histological sections of 3 μ m were slightly shallow etched with half-saturated sodium hydroxide solution for 20 min and were subsequently washed several times with phosphate buffered saline (PBS, PAA Laboratories GmbH). To minimize non-specific binding, samples were incubated www.nature.com/scientificreports/ Scientific Reports | 6:35606 | DOI: 10.1038/srep35606 11 45 min with donkey serum (Jackson Immuno Research Laboratories Inc.) including 0.15% Triton-X-100 (Sigma-Aldrich Chemie GmbH). Afterwards, slices were incubated for 60 min with following primary antibodies: Anti-smooth muscle actin (ready to use, Zytomed Systems GmbH) and anti-purinergic receptor P2X7 (1:25, Sigma Aldrich Chemie GmbH). As negative controls, the primary antibodies were replaced by bovine serum albumin (BSA Serva Electrophoresis GmbH). Following the repeated rinsing with PBS, histological slices were incubated for further 60 min with secondary antibodies consisting of goat anti-mouse IgG-Cy3 (1:200, Invitrogen) and goat anti-rabbit IgG-Cy2 (1:200, Jackson Immuno Research Laboratories Inc.). Additionally, slices were incubated for 2 h with tetramethylrhodamine B isothiocyanate/phalloidin (1:500, Sigma-Aldrich Chemie GmbH). After antibody staining, all slices received staining of nucleus through 4′,6-diamidino-2-phenylindole/DAPI (1:5000, Invitrogen) by an incubation time of 10 min and were embedded with the same silicone oil mixture that was used for SLOT imaging. All incubation steps were performed at room temperature. Immunofluorescence sections were recorded at 10× magnification with a Zeiss AxioScan.Z1.

### TEM

In order to obtain slices of the CRISTAL embedded samples for correlative TEM, serial sections of 15 μm slices were prepared. After ROIs were identified via toluidine blue staining 2 mm x 2 mm regions were cut out with a scalpel, incubated in EPON (Agar 100 resin, Agar Scientific) at 40 °C for 1 h and mounted on EPON dummies via incubation at 40 °C for 24 h. Finally, the samples with the EPON dummies were completely cured at 80 °C for further 48 h. Subsequently, the resin blocks were trimmed and 60 nm ultrathin sections were cut. After poststaining with uranyl acetate and lead citrate (staining step in saturated uranyl acetate solution in 75% methanol, 5 min; washing steps with 70% and 30% methanol, 30 s each; 2x distilled water, 5 min each; lead citrate according to Reynolds^39^), samples were analyzed with a TEM system (Morgagni, FEI) equipped with a 2 K side mounted Veleta CCD camera (Olympus Soft Imaging Solutions GmbH). Images were processed by adapting contrast and sharpness.

## Additional Information

**How to cite this article**: Kellner, M. *et al.* A combined method for correlative 3D imaging of biological samples from macro to nano scale. *Sci. Rep.* **6**, 35606; doi: 10.1038/srep35606 (2016).

## Supplementary Material

Supplementary Information

Supplementary Video 1
