# Supplementary material for: A combined method for correlative 3D imaging of biological samples from macro to nano scale
Source: Sci Rep. 2016 Oct 19;6:35606. doi: 10.1038/srep35606 (PMC5069670; doi:10.1038/srep35606)
Supplement: Supplementary Information [file srep35606-s1.pdf]

A combined method for correlative 3D imaging of biological samples from macro to nano scale

Manuela Kellner[1,5,*], Marko Heidrich[2,*], Raoul-Amadeus Lorbeer[2], Georgios C. Antonopoulos[2], Lars Knudsen[1,5], Christoph Wrede[1,5,6], Nicole Izykowski[3,5], Roman Grothausmann[1,6], Danny Jonigk[3,5], Matthias Ochs[1,5,6], Tammo Ripken[2], Mark P. Kühnel[1,5,6,+], Heiko Meyer[2,4,+]

[1] Institute of Functional and Applied Anatomy, Hannover Medical School, Hannover, Germany

[2] Biomedical Optics Department, Laser Zentrum Hannover e.V., Hannover, Germany

[3] Institute for Pathology, Hannover Medical School, Hannover, Germany

[4] Department of Cardiothoracic, Transplantation and Vascular Surgery (HTTG), Hannover Medical School, Hannover, Germany

[5] Biomedical Research in Endstage and Obstructive Lung Disease Hannover (BREATH), Member of the German Center for Lung Research (DZL), Hannover, Germany

[6] REBIRTH Cluster of Excellence, Hannover Medical School, Hannover, Germany

* These authors contributed equally to this study and share first authorship.

+ These authors contributed equally to this study and share senior authorship.

Correspondence: Heiko Meyer, h.meyer@lzh.de

Supplementary Information

**Supplementary Figure 1**	CRISTAL sample preparation
**Supplementary Figure 2**	Transmission characteristics and penetration depth of CRISTAL polymer mixture
**Supplementary Figure 3**	MPM measurement of the healthy rat control lung lobe and correlation with SLOT imaging
**Supplementary Figure 4**	Toluidine blue staining of the healthy control lung and correlation with SLOT imaging
**Supplementary Figure 5**	Correlative TEM analysis of in SLOT selected areas in a healthy lung.
**Supplementary Figure 6**	Immunofluorescence analysis of healthy control lung
**Supplementary Figure 7**	SLOT imaging of mouse brain CRISTAL sample
**Supplementary Figure 8**	SLOT imaging of mouse paw CRISTAL sample

Supplementary Figure 1 | CRISTAL sample preparation

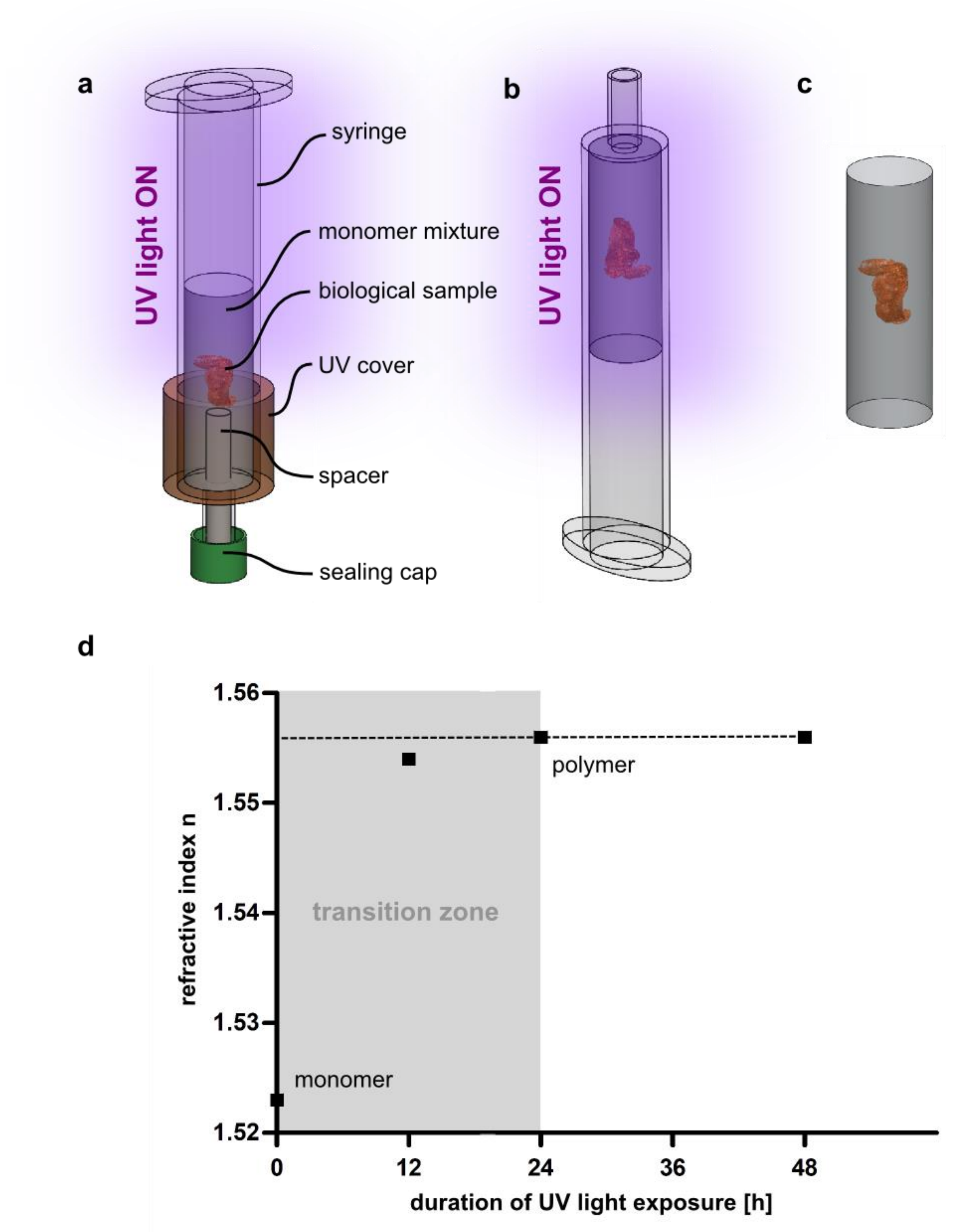

Supplementary Figure 1 | CRISTAL sample preparation. (**a**) First, the previously fixated and dehydrated sample is placed in a syringe filled with the monomer mixture and exposed for several minutes to UV light at a temperature of 4°C. Cooling during the preparation process is crucial, since the polymerization produces heat that has to be dissipated. The sample is positioned on a spacer to prevent it from subsidence in the syringe. A UV cover cap at the lower part of the syringe ensures that almost only the upper half of the resin is allowed to polymerize. (**b**) After that, the spacer is removed, the syringe is turned upside down and the entire sample is further polymerized under UV light at 4°C for 24 h. (**c**) Finally, complete polymerization is achieved, the syringe is removed and the prepared CRISTAL sample is available for SLOT imaging. (**d**) The time dependent refractive index measurement of the polymer indicates a complete polymerization of the resin after 24 h, when the CRISTAL block reaches the final refractive index of 1.556.

Supplementary Figure 2 | Transmission characteristics and penetration depth of CRISTAL polymer mixture

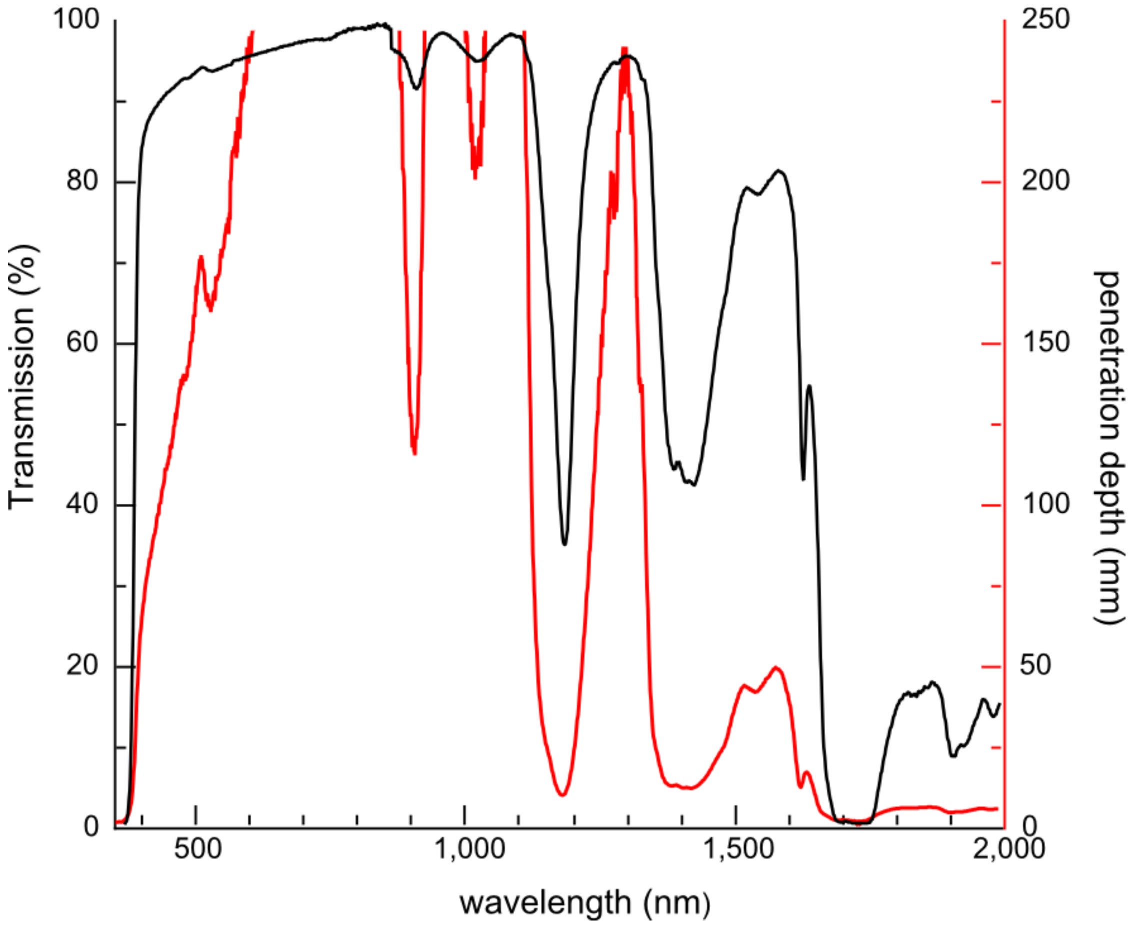

Supplementary Figure 2 | Transmission characteristics and penetration depth of CRISTAL polymer mixture. Analysis of the transmission of CRISTAL polymer block of 10 mm thickness in the spectral range of 350 nm to 2000 nm. The polymer consists of the 1 part NOA 68 and 7 parts NOA 71 monomers used for the CRISTAL clearing. The transmission curve (black) was normalized to compensate for the attenuation due to reflections and scattering on the surface of the test block. The polymer is apparently completely clear and colorless in the visible range beginning at 400 nm with dips in the near infrared at 900 nm and 1185 nm. Penetration depth (red) was calculated as the depth at which the intensity is extenuated to 1/e (= 37.78%) of the input intensity. Thus, common UV-emitting LEDs for polymerization yield penetration depths between 1.8 mm (at 365 nm) and 61 mm (at 395 nm), respectively.

Supplementary Figure 3 | MPM measurement of the healthy rat control lung lobe and correlation with SLOT imaging

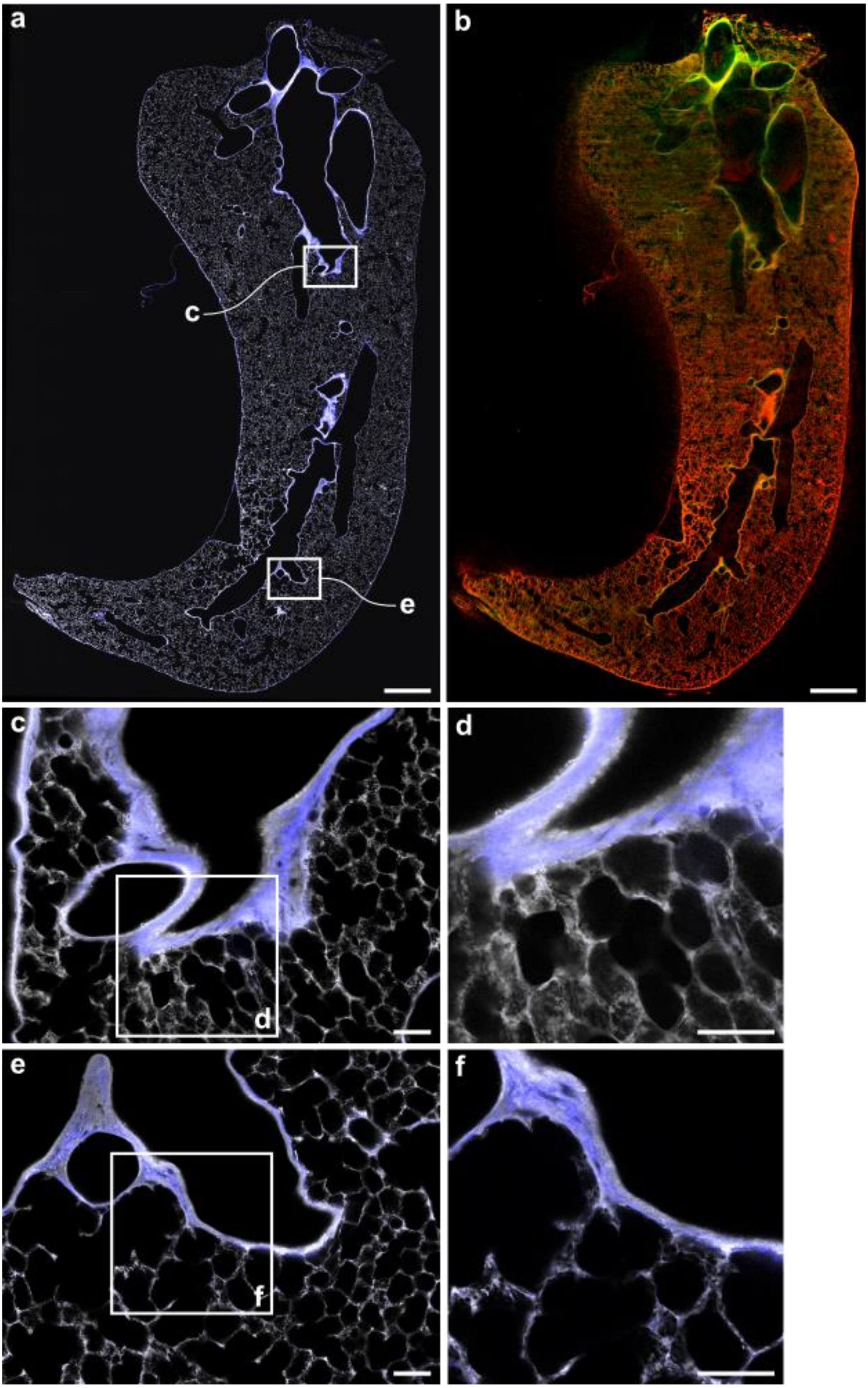

Supplementary Figure 3 | MPM measurement of the healthy rat control lung lobe and correlation with SLOT imaging. (**a**) 2D mosaic MPM visualizing autofluorescence (white) at 525 nm $\pm$ 25 nm excited at 850 nm and SHG (blue) signal at 425 nm in a depth of 280 µm below the cut surface of the CRISTAL sample. The mosaic consists of 25 x 40 tiles each covering 350 µm x 350 µm resulting in a total area of 8.75 mm x 14 mm of the shown slice. (**b**) Corresponding reslice through the SLOT 3D data stack acquired prior to sectioning and MPM measurements. Autofluorescence (green) at 570 nm LP excited at 532 nm is merged with signal of the transmission channel representing absorption (red). Since SLOT imaging covers the entire volume of the rat control lung (here approx. 2200 mm^3) with a resolution at the alveolar level, reslicing can be performed at any preferred direction in the sample. That allows fast screening of the complete sample prior to further analyzing steps with additional imaging techniques in the same CRISTAL sample. Thereafter, SLOT enables their spatial correlation by providing 3D and isotropic accessibility in the complete sample. (**c**, **e**) Enlarged views of the boxed regions in **a**. (**d**, **f**) Enlarged views of the boxed regions in **c** and **e**. In contrast to the bleomycin-treated lung, no increased collagen deposits in the parenchyma could be observed. However, collagen and elastin is universally present especially in blood vessels and bronchial walls. Scale bars, 1 mm (**a, b**), 100 µm (**c, d, e, f**).

Supplementary Figure 4 | Toluidine blue staining of the healthy control lung and correlation with SLOT imaging

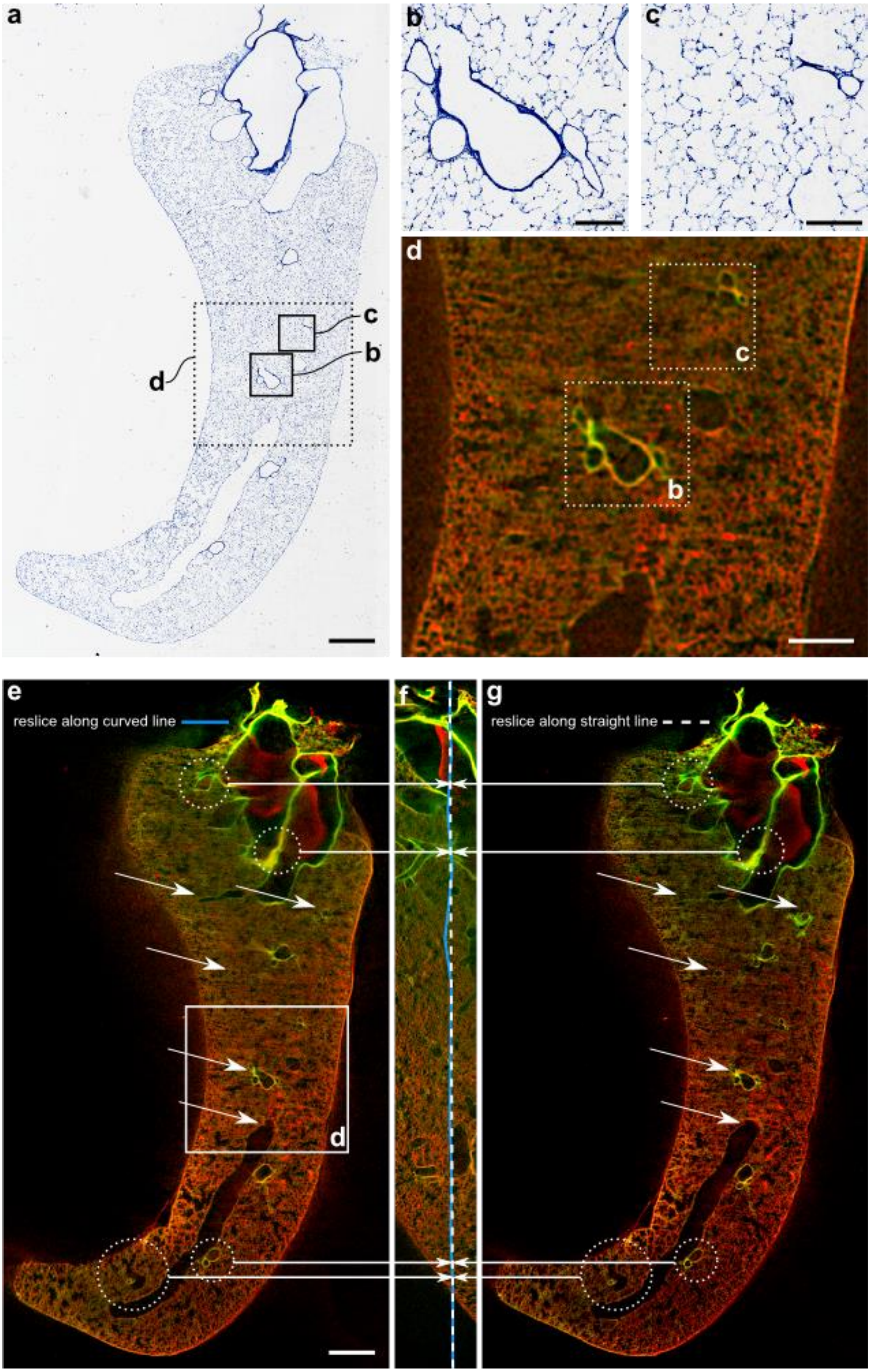

Supplementary Figure 4 | Toluidine blue staining of the healthy control lung and correlation with SLOT imaging. (**a**) Bright field analysis of histological section stained with toluidine blue. The cutting direction was along the long side of the illustration. (**b, c**) Enlarged views of the boxed regions in **a**. (**d**) Enlarged view of the SLOT reslice shown in **e** corresponding to the large boxed region in **a**. Red indicates absorption at 532 nm measured with the transmitted light channel. Green visualizes autofluorescence at 570 nm LP. The boxes correspond to the enlarged view in **b** and **c**, respectively. (**e**) Orthogonal reslice through the 3D SLOT data stack along the curved line (blue) in the orthogonal reslice shown in **f**. The curved reslice in **e** has a better agreement with the histological section in **a** than the reslice along a straight line. Agreement with the histological section is given in both reslices (**e** and **g**) for the encircled regions. In contrast, the regions indicated by the arrows in **e** and in **g** only show agreement with the histological sections for the curved reslice. This points to slight elevation and depression of the knife in the order of 30 µm during the cutting process. Moreover, a slight shrinkage of 94.1% in the direction of cutting in the histological section compared to the SLOT reslice is observable. Scale bars, 1 mm (**a, e**), 500 µm (**d**), 200 µm (**b, c**).

Supplementary Figure 5 | Correlative TEM analysis of in SLOT selected areas in a healthy lung.

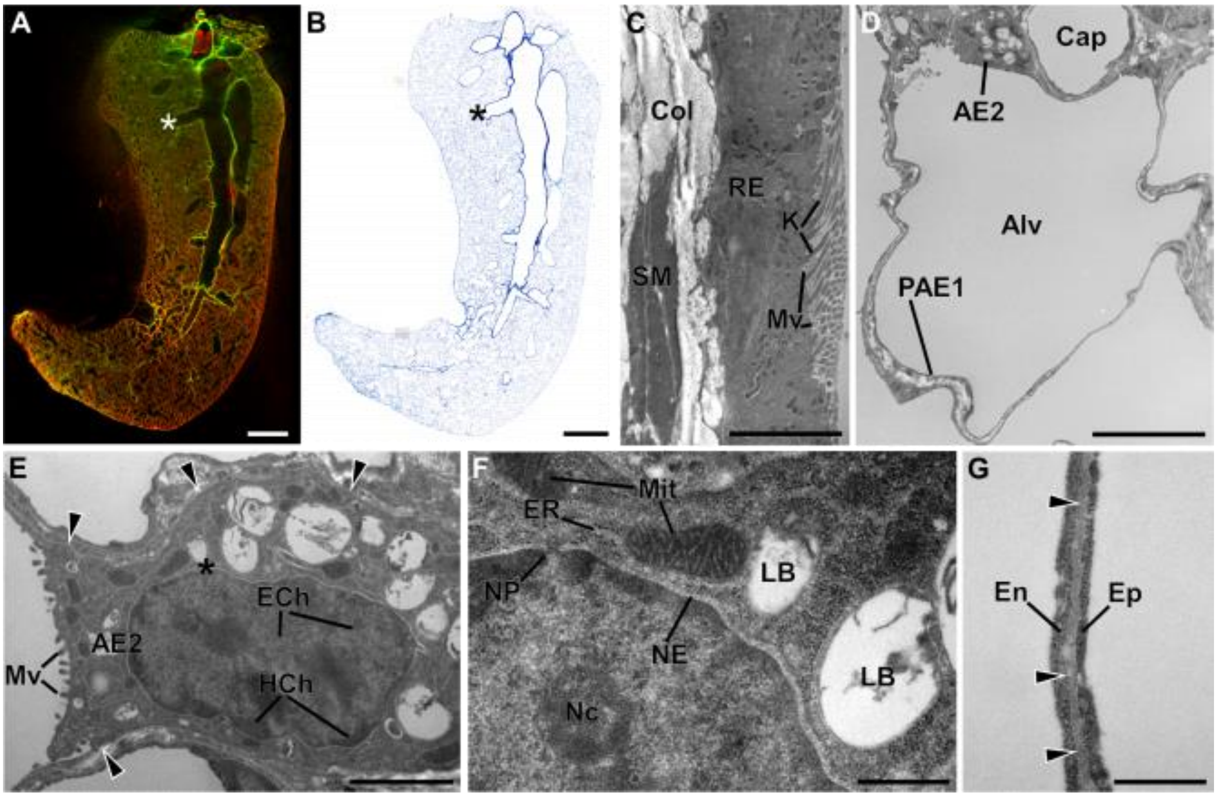

Supplementary Figure 5 | Correlative TEM analysis of areas in a healthy lung selected in SLOT. (**a**) Curved reslice in SLOT 3D data stack (**Supplementary Fig. 4f**) for correlation with histological section in **b** and region of TEM analysis. (**b**) Histological section of targeted area in the lung. The TEM analyzed area is marked with asterisk. (**c**) Electron microscopy image of conducting airway. Respiratory epithelium (RE) with kinocilia (K) and microvilli (Mv), smooth muscle (SM) and surrounding collagen (Col) are depicted. (**d**) Lung parenchyma with alveolus (Alv). Protrusions of alveolar epithelial type I (PAE1) and alveolar epithelial type II cells (AE2) are lining the alveolus. Together with capillaries (Cap), the blood-air barrier is formed. (**e**) Alveolar epithelial type II cell (AE2) with microvilli located on the alveolar epithelial basal lamina (arrow heads). Eu- and Heterochromatin (ECh/HCh) are visible. (**f**) High magnification of an alveolar epithelial type II cell (at asterisk in **e**). Details like ribosomes, endoplasmic reticulum (ER), mitochondria with cristae (Mit), lamellar bodies (LB, lamella mostly extracted), nucleolus (Nc) and nuclear envelope (NE) with nuclear pore (NP) are visible. (**g**) Blood-air barrier composed of endothelium (En), basal lamina (arrow heads) and epithelium (Ep). Scale bars, 1 mm (**a**, **b**), 5 µm (**c**, **d**), 2 µm (**e**), 0.5 µm (**f**, **g**).

Supplementary Figure 6 | Immunofluorescence analysis of the healthy control lung.

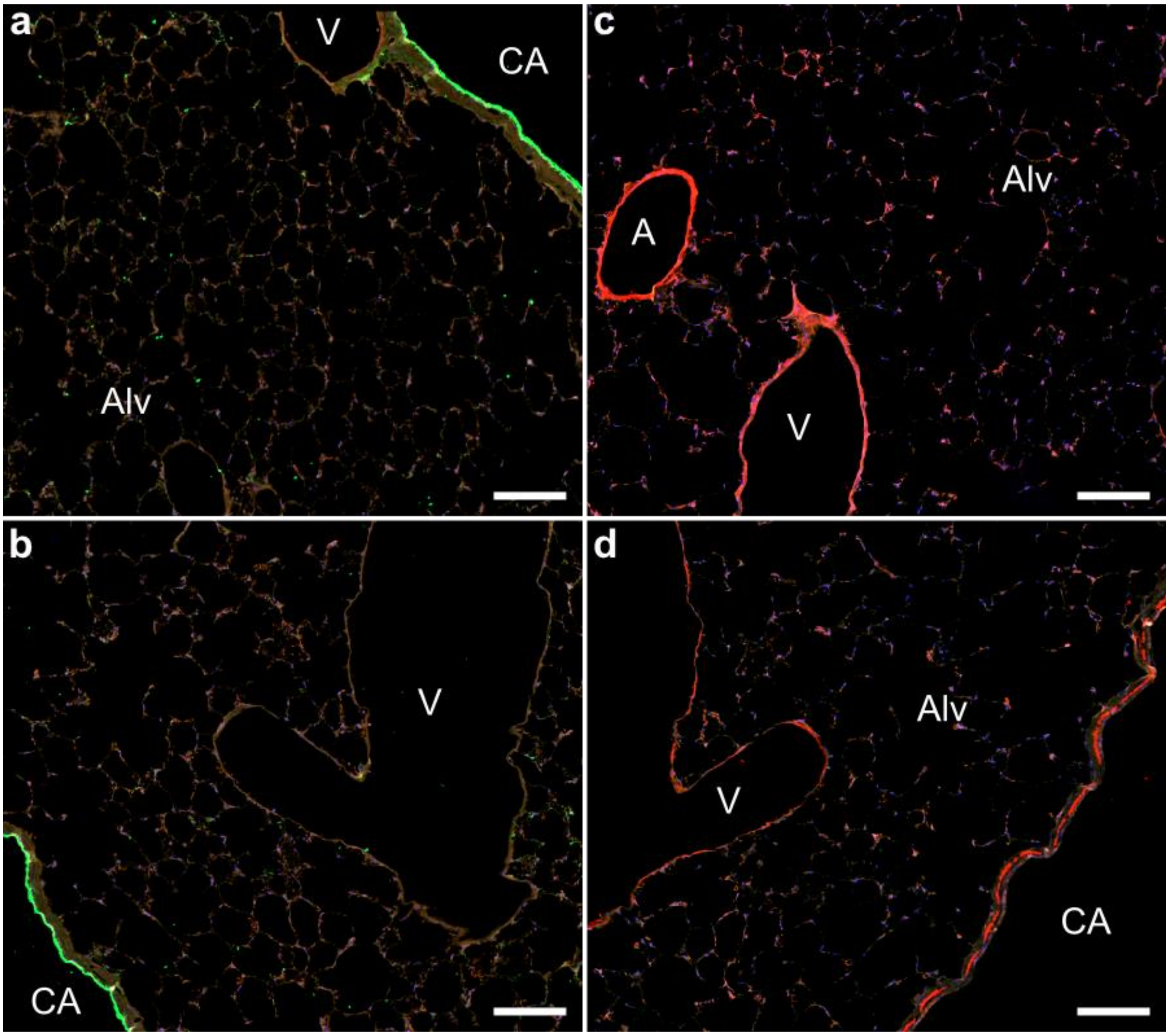

Supplementary Figure 6 | Immunofluorescence analysis of the healthy control lung. (**a, b**) Immunofluorescence of the purinergic receptor P2X7 reveals intense staining of bronchial epithelia (green), (**c, d**) Immunofluorescence of smooth muscle actin in walls of conducting airways and blood vessels, as well as individual cells in the parenchyma (red). (Conducting airways (CA), veins (V), arteries (A), alveoli (Alv)). Scale bars, 100 µm (**a-d**).

Supplementary Figure 7 | SLOT imaging of mouse brain CRISTAL sample

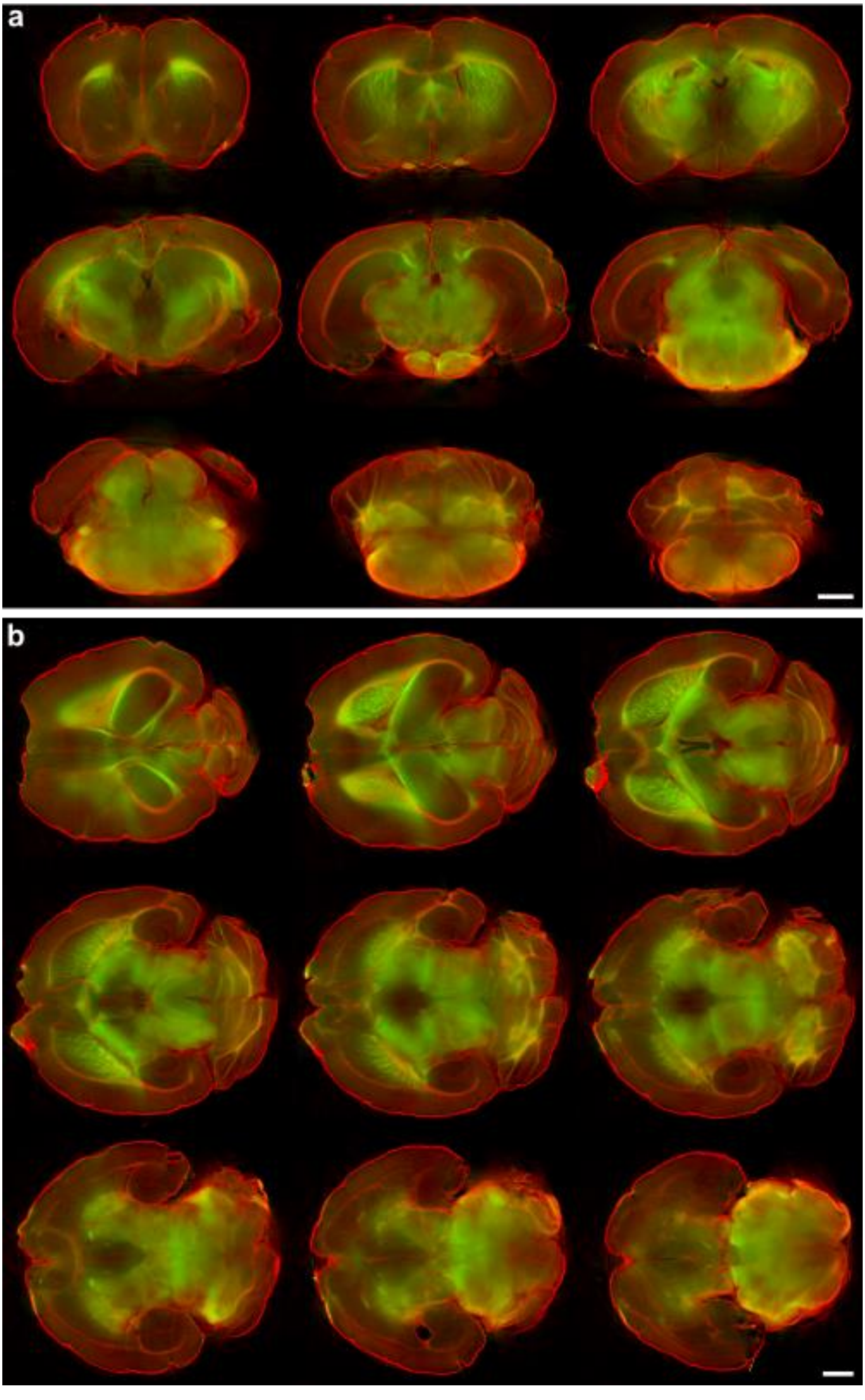

Supplementary Figure 7 | SLOT imaging of a mouse brain CRISTAL sample. Reslices of merged transmission (red) and scattered light (green) channel through 3D SLOT data stack. (**a**) Reslices in steps of 777 µm in dorso-ventral direction. (**b**) Reslices in steps of 310 µm in horizontal direction. Via endogenous contrast mechanisms structures like cerebrum and cerebellum can be identified. Additionally, inner structures like nucleus lentiformis and corpus callosum are visible. Scale bars, 1 mm (**a, b**).

Supplementary Figure 8 | SLOT imaging of mouse paw CRISTAL sample

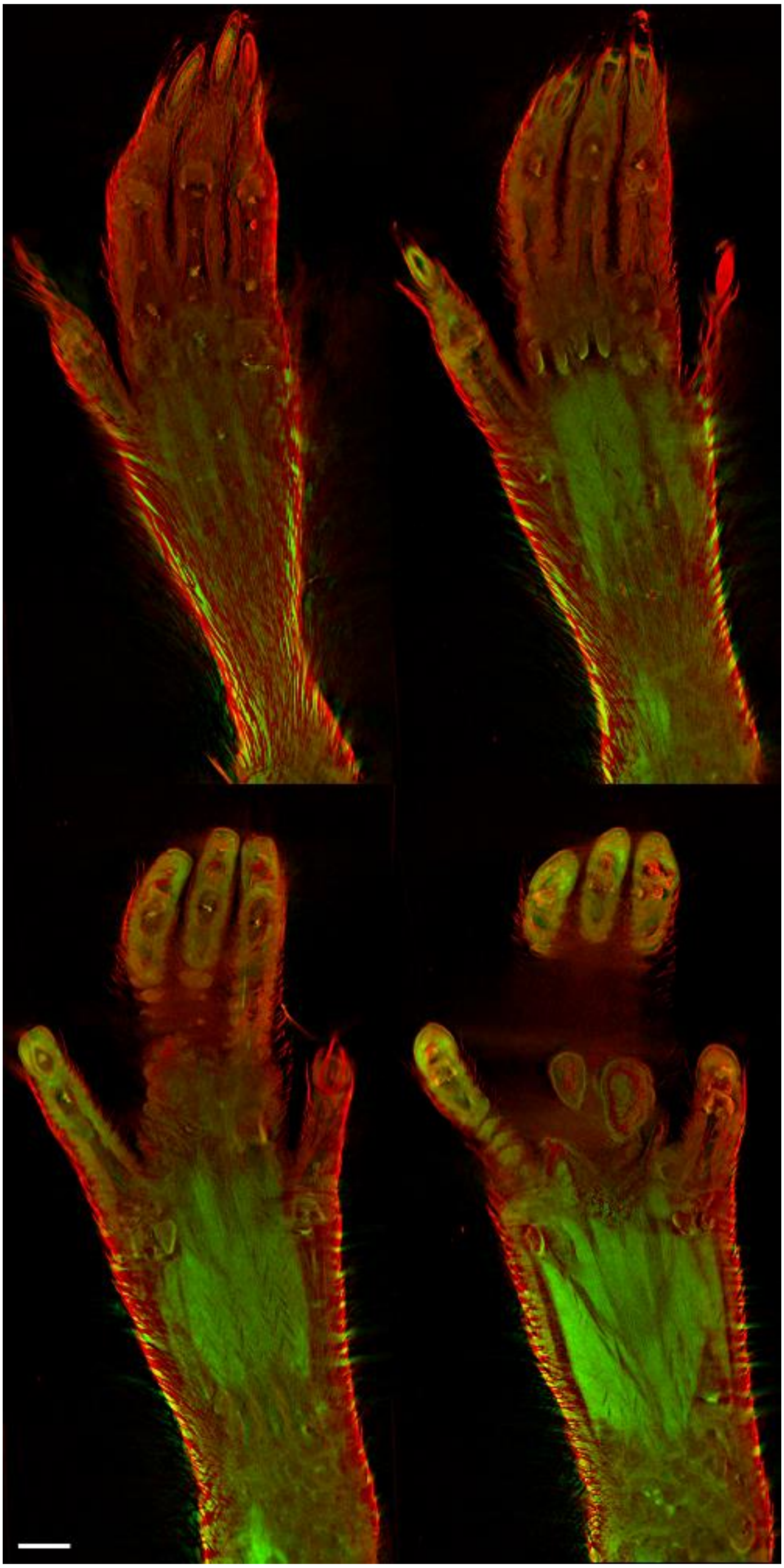

Supplementary Figure 8 | SLOT imaging of mouse paw CRISTAL sample. The sample was prepared according to the CRISTAL method and subsequently imaged with SLOT. Transmission (red) and autofluorescence (green) channel are merged in reslices through the volumetric data stacks in steps of 384 µm. Via endogenous contrast mechanisms the bone composition of the mouse claw including articular cartilage and medullary cavity are detectable. Scale bar 1 mm.
